# Cytological, Physiological, and Transcriptome Analysis of Leaf-Yellowing Mutant in *Camellia chekiangoleosa*

**DOI:** 10.3390/ijms26010132

**Published:** 2024-12-27

**Authors:** Bin Huang, Wenyin Huang, Zhenyu Liu, Yixuan Peng, Yanshu Qu, Wencai Zhou, Jianjian Huang, Huili Shu, Qiang Wen

**Affiliations:** 1 Jiangxi Provincial Key Laboratory of Oil-Tea Camellia Resource Cultivation and Utilization, Jiangxi Academy of Forestry, Nanchang 330032, China; huangbin007@yeah.net (B.H.); 13970957560@163.com (W.H.); 18811716268@163.com (Y.P.); ysqu@jxlky.cn (Y.Q.); zhouwencai2000@163.com (W.Z.); huang9931@126.com (J.H.); 18970803609@189.cn (H.S.); 2 College of Forestry, Jiangxi Agricultural University, Nanchang 330045, China; m15396981126@163.com

**Keywords:** *Camellia chekiangoleosa*, leaf-yellowing mutant, physiological, cytological, transcriptome, co-expression regulatory network

## Abstract

Color variation in plant leaves has a significant impact on their photosynthesis and plant growth. *Camellia chekiangoleosa* yellow-leaf mutants are ideal materials for studying the mechanisms of pigment synthesis and photosynthesis, but their mechanism of leaf variation is not clear. We systematically elucidated the intrinsic causes of leaf yellowing in the new *Camellia chekiangoleosa* variety ‘Diecui Liuji’ in terms of changes in its cell structure, pigment content, and transcript levels. This study indicates that the incomplete structure of chloroplast-like vesicles, the decrease in blue-green chlorophyll a, and the increase in yellow-green chlorophyll b in yellowing leaves are the direct causes of yellowing-leaf formation. The high expression of genes that catalyze the degradation of chlorophyll a (*PAO* and *RCCR*) and its conversion to chlorophyll b (*CAO*) in yellowing leaves leads to a decrease in the chlorophyll a content, while the low expression of *CLH* genes is the main reason for the increase in the chlorophyll b content. We also found transcription factors such as ERF, E2F, WRKY, MYB, TPC, TGA, and NFYC may regulate their expression. RT-qPCR assays of 12 DEGs confirm the RNA-seq results. This study will provide a foundation for investigating the transcriptional and regulatory mechanisms of leaf color changes.

## 1. Introduction

Leaf color variation is a common phenotypic feature of plants and is widespread in nature. Leaf color variation in horticultural plants has certain ornamental and economic values and is an important source of new germplasm selection and even hybrid breeding. Stable leaf color variation is often accompanied by physiological and biochemical processes and regulation at the molecular level, such as plastid development, photosynthetic system assembly and operation, and pigment metabolism [1]. Therefore, leaf color mutants are ideal materials for studying the mechanisms of leaf color regulation, photosynthesis mechanisms, and pigment metabolic pathways in plants [2]. In recent years, more and more studies have focused on the link between leaf color variation and environmental adaptation and even resistance. It can be seen that the study of the mechanism of leaf color variation is an important topic in the field of plant development and genetic breeding [3].

The leaf color of plants is determined by the content, percentage, and type of pigments. The leaves of higher plants mainly contain three types of pigments: chlorophyll, carotenoids, and anthocyanins. Among them, chlorophyll is the core pigment of photosynthesis and is divided into chlorophyll a and chlorophyll b, which are blue-green and yellow-green, respectively [1]. The chlorophyll metabolic pathway in higher plants consists of three steps: chlorophyll biosynthesis, the chlorophyll cycle (the interconversion of chlorophyll a and chlorophyll b), and chlorophyll degradation [4,5,6]. Carotenoids are a collective term for carotenoids and lutein, which are terpenoids. Carotenoids are yellow and orange in color and are essential for protecting plants for normal photosynthesis [7]. Cis-lycopene (lycopene), carotenoids, and lutein are the major carotenoids in the plant photosystem. Anthocyanins mainly exist in the form of anthocyanins, which are a subclass of flavonoids and secondary products in plant metabolism. They are widely distributed in various organs of plants, with over 550 known species, most of which are derived from the combination of six basic anthocyanins (geranium pigments, swiftgrass pigments, cornflower pigments, morning glory pigments, peony pigments, and mallow pigments) with different glycosides [8]. Previous studies have shown that the formation of red and purple leaves in plants is dominated by anthocyanins [9], which have been reported in woody plants, such as *Acer rubrum* [10], delta poplar *Populus deltoides* [11], and *ginkgo biloba* [12], among others. The purple color variants ‘Zi Juan’, ‘Zi Yan’, and ‘Zi Chan’ in tea are representatives of anthocyanin accumulation [13]. The current research suggests that the yellow and white coloration of leaves is generally dominated by chlorophyll and carotenoids. For example, a yellow seedling lethal mutant, lrysl1, was found in the selfed progeny of *Lilium regale* by E. H. Wilson. The chlorophyll and carotenoid contents in the leaves of this mutant were lower than that of the wild type [14]. Su et al. determined the content of photosynthetic pigments in two leaf color compartments of six *Bambusoideae* species and found that the highest contents of pigments in the white and green regions were carotenoids and chlorophyll a, respectively, while the content of the two types of photosynthetic pigments in the white region was much lower than that in the green region [15]. Previous studies have also found that in the cucumber leaf color mutant C777 induced by EMS, the expression of chlorophyll-related genes (*CsHD*) is reduced in yellow leaves [16]. The molecular mechanism of yellow-leaf formation in plants is complex. For example, *Oryza sativa* yellow-leaf mutants exhibit abnormal chloroplasts [17]; the yellow-striped leaves of the *Cymbidium sinense* mutant are the result of increased chlorophyll degradation and metabolism [18]; the yellow-striped leaves of the *Lagerstroemia indica* yellow-leaf mutation is formed mainly due to differential expression of genes involved in chlorophyll biosynthesis and degradation [19]; and genes related to metabolism and carbon fixation are involved in leaf coloration in the yellow-white leaf mutants of *Camellia sinensis* [20].

As an important photosynthetic organ, leaf photosynthesis is the main source of carbon assimilation, providing energy for young leaves, roots, stems, flowers, fruits, and the developing seeds of plants. Especially in plants with fruits and seeds as economic organs, yield improvement depends on higher photosynthetic efficiency. Not only that, but leaves with excellent photosynthetic characteristics often have higher rates of photosynthesis, lower consumption of photosynthetic products, and greater adaptive capacity. Chlorophylls and carotenoids are responsible for absorbing light and transmitting excitation energy to reaction centers in the photosynthetic system, and Viljevac et al. showed that a decrease in photosynthetic pigments downregulates photosynthetic efficiency [21]. A T-DNA insertion was found within the promoter of the *OsAld-Y* gene in the rice chlorophyll-deficient mutant ygdl-1, resulting in low mRNA levels, and it was also demonstrated through transgenic complementation experiments that this gene is involved in chlorophyll accumulation, chloroplast development, and plant growth by affecting the photosynthetic rate and sugar metabolism in rice leaves [22]. *Camellia chekiangoleosa* Hu. has a narrow natural distribution system and is naturally distributed in the mountainous areas at an altitude of 400–1600 m at the junction of the provinces of Jiangxi, Fujian, and Zhejiang in China [23]. Its tree shape is full, its flowers are large and colorful, and its leaves are evergreen and broad, so it has great prospects for development in greening and coloring projects. As a rare stable diploid species in the genus *Camellia*, the research model of *Camellia chekiangoleosa* has great reference value for other species in the same genus. Our group has already deciphered its genome [24], which has laid an important foundation for investigating the molecular mechanism of the formation and regulation of *Camellia chekiangoleosa* traits. However, the mechanism of leaf coloration of *Camellia chekiangoleosa* is still unclear, which limits the utilization of its ornamental value and the understanding of its biological functions.

In this study, the molecular mechanism of foliage formation was systematically analyzed from its leaf cell structure, physiological changes, and gene expression profiles through transmission electron microscopy observation, physiological trait determination, and transcriptome sequencing using the leaves of ‘Diecui Liujin’ as experimental materials, with the aim of providing a molecular basis for the development of a new horticultural germplasm and even targeted genetic improvement in the future.

## 2. Results

### 2.1. Cytoarchitectural Changes in Mutant Leaves

**Figure 1:**
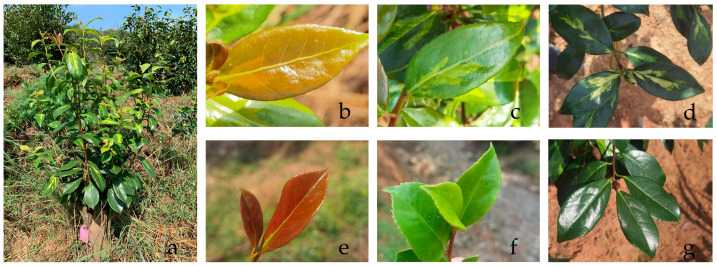
‘Diecui Liujin’ whole plant (**a**). Three periods were selected from young leaf formation to leaf maturity—10 DALFs (days after initial leaf formation, S1), 50 DALFs (S2), and 90 DALFs (S3)—for collection. S1–S3 period of yellowing leaves (**b**–**d**); S1–S3 period of normal leaves (**e**–**g**).

Since both the mutant leaves and the normal leaves were from the same plant and the same branch, there was no difference between the normal parts of the two. The young leaves were red, and as the leaves developed, the red color faded to a green color that deepened and deepened until the leaves reached full maturity and remained dark green. The only difference was the yellowing of the mutant leaves from the petiole and along the main veins, which changed to a distinct yellow color as the leaf expanded (Figure 1).

**Figure 2:**
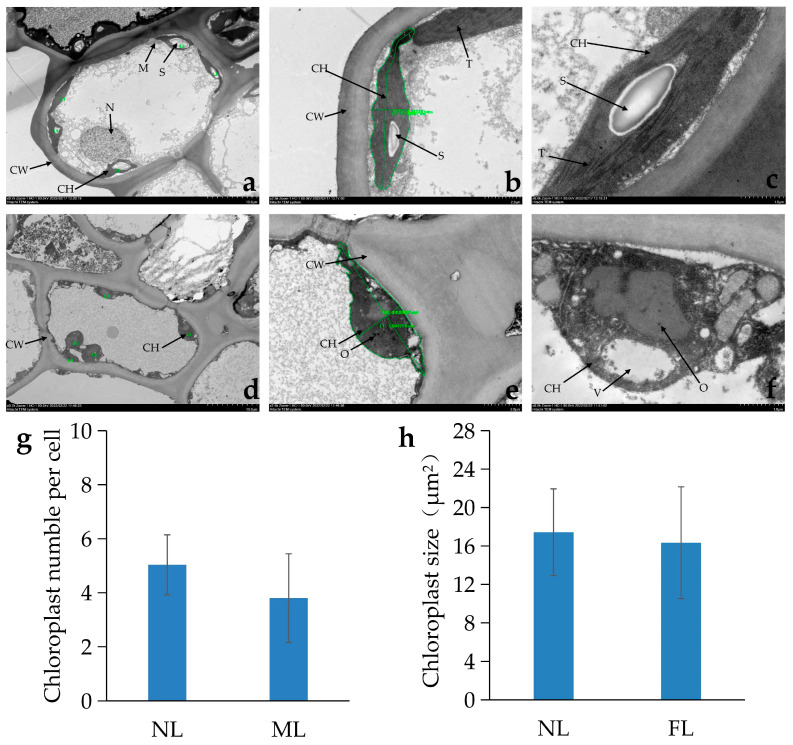
Chloroplast ultrastructure on normal green leaves and mutant leaves of *C. chekiangoleosa*. Bars = 10 μm (**a**,**d**) and 2 μm (**b**,**c**,**e**,**f**). S, starch granules; M, mitochondrion; N, nucleus; CH, chloroplast; CW, cell wall; T, thylakoid grana; O, osmiophilic granules; V, vesica; NL, normal leaf; ML, mutant leaf. The average number of chloroplasts per cell (**g**) and mean chloroplast size were lower in the mutant leaves (**h**).

Through transmission electron microscopy, we further observed the ultrastructure of the cells and even the chloroplasts and found that the chloroplast structures of the yellowed part and the normal leaves were significantly different. The chloroplasts in normal leaves (Figure 2a–c) showed obvious thylakoid membranes. We observed the typical thylakoid grana structure with obvious starch granules (usually 1~2 granules). On the contrary, the membrane structure of chloroplasts in the yellowing part of chloroplasts was blurred, and the cyst-like lamellar structure of the thylakoid grana was not observed. A few chloroplasts gradually fell off from the attachment of the cell membrane (Figure 2d), there was no obvious starch granule formation, and some vesicles and a few osmiophilic granules appeared (Figure 2d–f). In terms of the average number and area of chloroplasts per cell, normal cells had, on average, one more chloroplast and a slightly higher chloroplast area, but there was no significant difference (Figure 2g,h). In conclusion, the phloem indicated that the destruction of the chloroplast structure may be the main reason for the formation of the yellowing part and the phenomenon of the phloem.

### 2.2. Changes in Photosynthesis of Mutant Leaves

**Figure 3:**
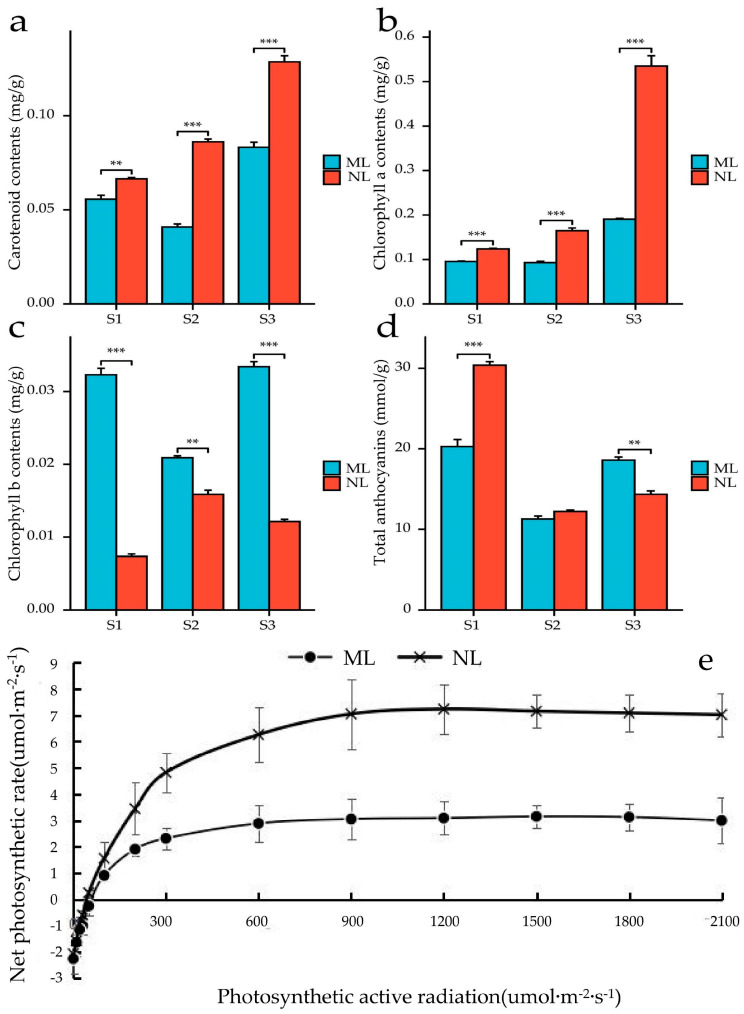

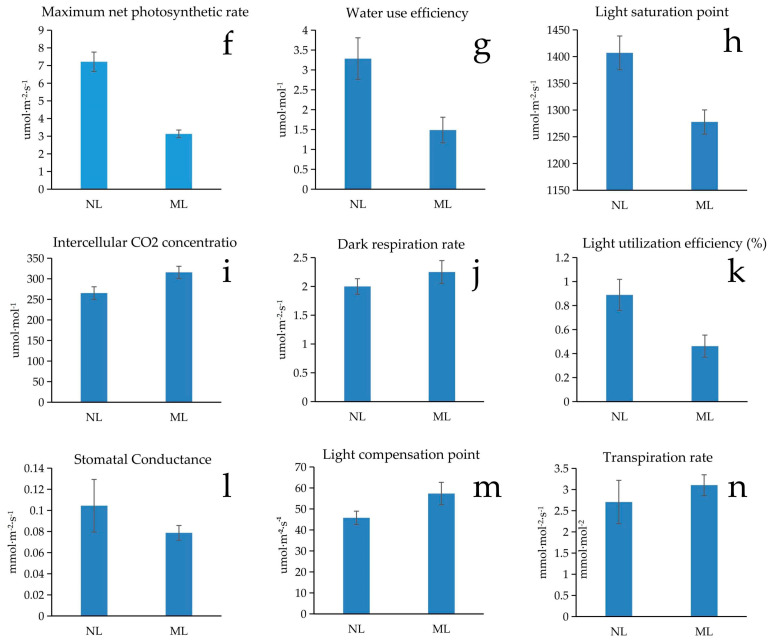
Changes in pigment content of normal leaves and mutant leaves (**a**–**d**). Changes in photosynthetically active radiation of normal leaves and mutant leaves (**e**). Changes in photosynthetic parameters of normal leaves and mutant leaves (**f**–**n**). S1, Stage 1; S2, Stage 2; S3, Stage 3. **, *p* < 0.01; ***, *p* < 0.001.

Changes in the structure of the chloroplasts in cells often have important effects on photosynthesis in plants. In order to clarify the effect of changes in the cell structure on plant growth, this study measured the photosynthetic indexes of mutant leaves and normal leaves. Using line graphs, it was found that the photosynthesis of normal leaves was significantly stronger than that of flower leaves (Figure 3e), with the maximum photosynthesis rate of normal leaves reaching 7.2 umol·m^−2^·s^−1^, which was more than twice as much as that of mutant leaves at 3.13 umol·m^−2^·s^−1^. The light saturation point, water-use efficiency, and light-energy utilization rate of normal leaves were also significantly higher than that of mutant leaves (Figure 3f–h,k). In addition to this, the light compensation point, dark respiration point, and transpiration of normal leaves were slightly lower than that of the flowering leaves, whereas the stomatal conductance of the mutant leaves was lower than that of normal leaves, and less CO_2_ entered the cells, resulting in a higher concentration of intercellular CO_2_, which also contributed to the weakening of photosynthesis. Combined with the changes in the chloroplast structure, this suggests that the changes in the chloroplast structure are the main reason for the decline in photosynthesis in mutant leaves, accompanied by decreases in stomatal conductance, water utilization, and photosynthetic efficiency.

### 2.3. Metabolite Changes in Mutant Leaves

**Figure 4:**
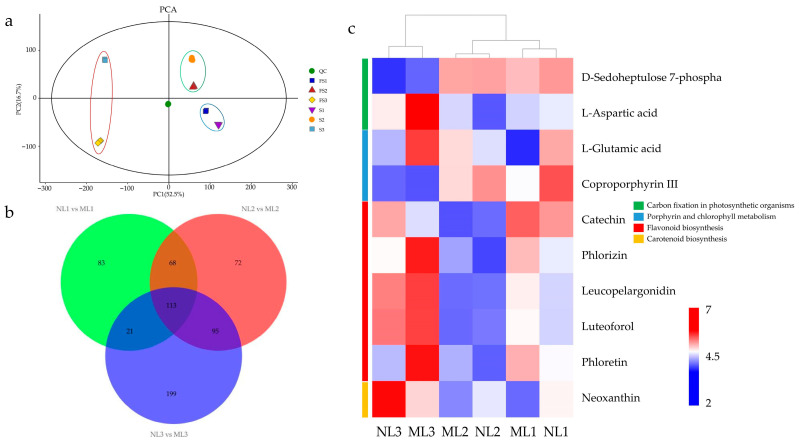
PCA plots of non-targeted metabolomes of six sets of samples (**a**), Venn plots of differential metabolites of two leaf types at three developmental periods (**b**), and heat maps of relative metabolite contents of the pathways of interest (**c**). NL1, Stage 1 of normal leaf; NL2, Stage 2 of normal leaf; NL3, Stage 3 of normal leaf; ML1, Stage 1 of mutant leaf; ML2, Stage 2 of mutant leaf; ML3, Stage 3 of mutant leaf.

In order to comprehensively investigate the changes in the metabolic process of mutant leaves, this study obtained non-targeted metabolomic data from normal and mutant leaves. A total of 22,257 substance peaks were detected, and 2756 metabolites were obtained. Through the quality control (QC) of the samples, three biological replicates of six groups of samples were clustered together, indicating good experimental reproducibility, and two types of leaves in the same period were also clustered together, reflecting the scientific nature of the sampling (Figure 4a). A comparison of the metabolites from two kinds of leaves in the same period showed that 285, 348, and 428 differential metabolites (DEMs) were detected in NL1: ML1, NL2: ML2, and NL3: ML3, respectively, with a total of 113 differential metabolites in the three-group comparison (Figure 4b). Screening from the co-differential metabolites in the three-group comparison reveals that D-sedoheptulose 7-phosphate and L-aspartic acid were involved in carbon fixation in photosynthesis; L-glutamic acid and Coproporphyrin III were involved in porphyrin and chlorophyll metabolism; catechin, Phlorizin, Leucopelargonidin, Lutroforol, and Phloretin were metabolites in flavonoid metabolism, and Neoxanthin was a precursor of lutein biosynthesis in carotenoid metabolism (Figure 4c). L-glutamic acid is a reaction substrate of photosynthesis, and Coproporphyrin III is an intermediate product, both of which accumulate more in yellowed leaves, suggesting that chlorophyll synthesis may not be the main cause of the yellowing phenomenon, pending further analysis of the causes at the physiological and molecular levels.

### 2.4. Pigmentation Changes in Mutant Leaves

To further analyze the direct cause of mutant leaf formation, the present study detected and analyzed the pigment content changes in normal and mutant leaves (Figure 3a–d). During the development of leaves, except for chlorophyll b, the trend of pigment changes in normal leaves and yellowed parts was basically the same. Carotenoids and chlorophyll a both increased during development and reached their highest at the S3 stage, while total anthocyanins appeared to decrease first and then increase a little. While chlorophyll b in normal leaves increased first and then decreased, the opposite was true for the yellowed parts, in which it decreased first and then increased and was the highest at the S3 stage. Comparing the pigment contents of normal leaves and the yellowed portion of the leaves at each period, we found that they all showed consistency, i.e., the carotenoid content and chlorophyll a content were lower than those of normal leaves, the chlorophyll b content was higher than that of normal leaves, and the total anthocyanin content was lower than or approximate to that of normal leaves in the yellowed portion of the mutant leaves. The contents of important pigments related to leaf color (blue-green chlorophyll a and yellow-green chlorophyll b) in the two types of leaves were significantly different, especially the chlorophyll b content in yellowing leaves, which reached 0.031 mg/g in the S1 period and was about four times that of normal leaves (0.007 mg/g). The chlorophyll a content in normal leaves reached 0.535 mg/g in the S3 period, which was about three times that of yellowing leaves (0.191 mg/g). In conclusion, yellowing leaves have the highest chlorophyll a content, and its absence is accompanied by an increase in chlorophyll b, which is the direct cause of leaf yellowing.

### 2.5. Analysis of Expression Profile Changes and Differential Gene Annotation in Two Types of Leaves

**Figure 5:**
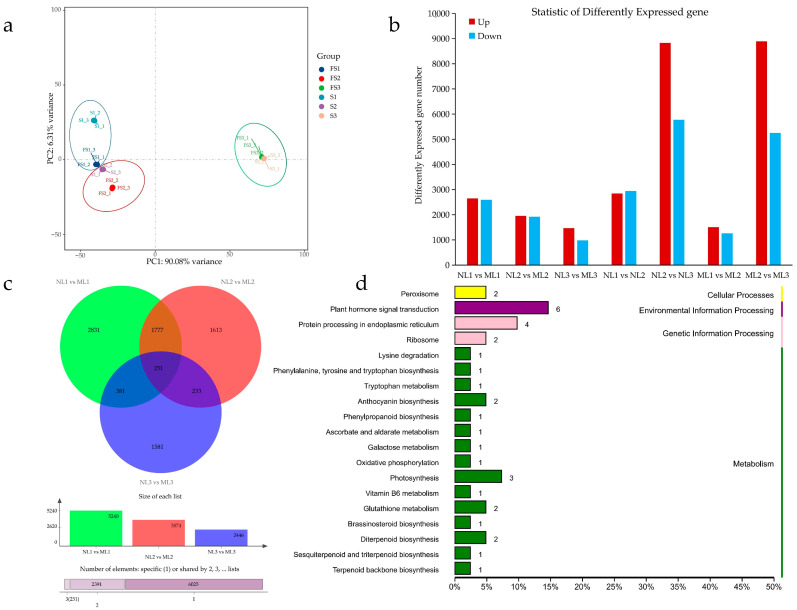
PCA plots of expression profiles of 18 samples (**a**), counts of differential genes among different samples (**b**), Venn plots and counts of differential genes of two leaf types at three developmental periods (**c**), and KEGG categorization plots of common differential genes of two leaf types at three developmental periods (**d**).

Based on the phenotypic, ultrastructural, and physiological changes described above, we speculated that the expression patterns of genes responsible for chloroplast development and pigment biosynthesis were altered in the mutants. To test our hypothesis, we performed transcriptomic comparisons. A total of 123.74 G of CleanData was obtained from the sequencing of the referenced transcriptomes of 18 *Camellia chekiangoleosa* samples in this study, and the effective data volume of each sample was distributed in the range of 6.4–7.35 G. The Q30 bases were distributed in the range of 94.54–95.05%, and the average GC content was 46.26%. Comparing the reads to the reference genome, the comparison rate was 90.55–95.14%. The PCA results based on gene expression show that the samples were better replicated, and the two types of leaves in the same period had higher similarity, indicating the accuracy of the sampling period (Figure 5a). Based on the comparison results, coding gene expression analysis was performed. Comparing the two types of leaves in the same period and different periods of the same leaf type, respectively, and screening for differentially expressed genes, it was found that the leaves had the most differentially expressed genes from the period of S2 to S3, which amounted to more than 14,000 in both normal and mutant leaves, far exceeding the other comparative groups, with the most active changes in the expression profiles; whereas, the differentially expressed genes in the normal and mutant leaves in the period of S1 were higher than those in the other two periods, reaching 5240, while the two leaves in the S3 period had the fewest differential genes, with only 2446 (Figure 5b,c).

In the comparison of the differences between the two types of leaves in the same period, the KEGG annotation of the differential genes between the two types of leaves in the S1 period (Supplemental Figure S1) to the most numerous classifications were protein processes in the endoplasmic reticulum, followed by plant hormone signal transduction, and the top 20 classifications in terms of the number of genes were flavonoid synthesis, porphyrin and chlorophyll synthesis, carbon fixation in photosynthetic organisms, photosynthetic antennae proteins, and photosynthesis. In S2 (Supplemental Figure S2), the KEGG classification of differential genes containing the most genes was also protein processes in the endoplasmic reticulum and plant hormone signal transduction, and the top 20 classifications were anthocyanin synthesis, porphyrin and chlorophyll synthesis, and photosynthesis. In S3, the two leaf differentials in the top 2 were phytopathogenic response and plant hormone signal transduction (Supplemental Figure S3), and the top 20 classifications were anthocyanin synthesis, flavonoid synthesis, and other pigment synthesis-related genes. Comprehensively, it was found that the differences in the assembly of enzymes, transcription factors, and other proteins in the endoplasmic reticulum were mainly concentrated in the first two periods. The expression of chlorophyll synthesis genes and photosynthesis-related genes was likewise concentrated in the first two periods, whereas the expression of flavonoid (including anthocyanin) synthesis genes was found to be throughout the entire period of leaf development. The differential expression of genes during leaf development reveals that the differential expression of chlorophyll synthesis and anthocyanin synthesis genes might be the main reason for the color change of leaves, and at the same time, the color change of leaves has an impact on the expression of photosynthesis-related genes, which leads to the decrease in photosynthesis in yellowed leaves.

In the comparison of the three groups, 251 genes were found to be differentially expressed (Figure 5d), and after KEGG enrichment classification, the most annotated category was plant hormone signal transduction. Among the six genes involved in plant hormone signal transduction, Cche03G001994 was annotated to the ORR9-like two-component response regulator, and its expression was higher in the S1 and S2 phases and lower in the S3 phase in normal leaves than in the mutant leaves, while the other five genes were annotated to the auxin response factor, auxin-responsive protein, and auxin transporter protein, respectively, which were all higher in the normal leaves, and among them, the auxin-responsive protein in the normal leaves was higher than the auxin-responsive protein in the S3 phase. Auxin-responsive protein was expressed at its highest in the S2 stage in both leaves, while the auxin response factor and auxin transporter protein were expressed at their highest in the S1 stage, followed by three photosynthesis-related genes (Cche13G002477, Cche04G004329, and Cche04G004330) and two anthocyanin synthesis genes (Cche02G000527 and Cche02G000520). The psaB protein encoded by Cche13G002477 is a key gene involved in photosynthesis system I. PetB encoded by Cche04G004329 is a subunit of the Cytochrome b complex, and the PsbB protein encoded by Cche04G004330 is also annotated to function as an electron-transferring protein in photosynthesis system II, both of which are downregulated in yellowing leaves. These three genes may be related to the difference in photosynthesis between the two leaf types, resulting in reduced photosynthesis in yellowing leaves. Cche02G000527 and Cche02G000520 both encode kaempferol 3-O-β-D-galactosyltransferases, which are involved in anthocyanin synthesis and upregulated in yellowing leaves.

### 2.6. Expression of Senescence-Related Genes Was Not Upregulated in Yellowing Leaves

To determine whether leaf yellowing is related to leaf aging, this study compared the expression of senescence-related genes *SAGs* in two types of leaves (Supplemental Figure S4). It was found that the expression of all four *SAGs* with a certain amount of expression was slightly higher in normal leaves than in yellowed leaves, and the expression continued to increase with leaf growth and development, which was in accordance with the natural law. Moreover, no differential expression of hormones related to leaf senescence, such as gibberellin and abscisic acid, was found in the metabolomic data, which also ruled out the possibility that the formation of yellowed leaves was related to the intensification of leaf senescence.

### 2.7. Differential Expression Analysis of Chlorophyll Synthesis-Related Genes

**Figure 6:**
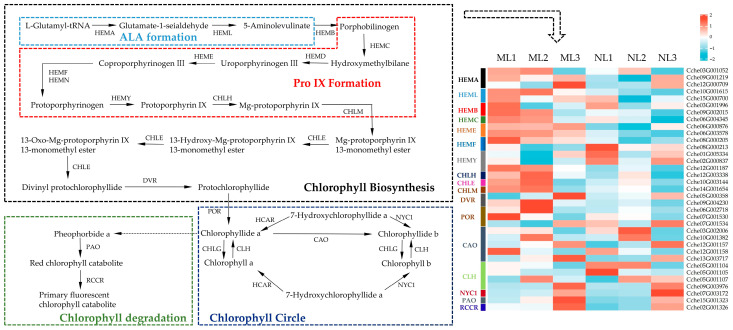
Differential expression of genes related to the chlorophyll metabolism pathway. The expression level was based on FPKM value. *HEMA*, glutamyl-tRNA reductase; *HEML*, glutamate-1-semialdehyde 2,1-aminomutase; *HEMB*, porphobilinogen synthase; *HEMC*, hydroxymethylbilane synthase; *HEMD*, Uroporphyrinogen-III synthase; *HEME*, uroporphyrinogen decarboxylase; *HEMF*, coproporphyrinogen-III oxidase; *HEMY*, oxygen-dependent protoporphyrinogen oxidase; *CHLH*, magnesium chelatase subunit H; *CHLE*, magnesium protoporphyrin IX monomethyl ester(oxidative) cyclase; *CHLM*, magnesium protoporphyrin IX methyltransferase; *DVR*, divinyl chlorophyllide a 8-vinyl-reductase; *POR*, protochlorophyllide reductase; *CAO*, chlorophyllide a oxygenase; *CLH*, chlorophyllase; *CHLG*, Chlorophyll synthase; *NYC1*, chlorophyll(ide) b reductase; *PAO*, pheophorbide a oxygenase; *RCCR*, red chlorophyll catabolite reductase.

The chlorophyll metabolic pathway in higher plants consists of three stages: chlorophyll synthesis, the chlorophyll cycle, and chlorophyll degradation (Figure 6). Changes in any of these three stages can lead to leaf yellowing. In addition, the metabolomic results show that Coproporphyrin III was less abundant in yellowed leaves, while glutamic acid accumulated significantly in both mid- and late-leaf development. Speculating that the chlorophyll metabolic pathway was affected in yellowed leaves, we focused on the core genes encoding the key enzymes involved in the chlorophyll metabolic pathway. We identified 35 differentially expressed candidate genes encoding 17 enzymes related to chlorophyll metabolism, of which 11 are involved in chlorophyll synthesis, 4 in the chlorophyll cycle, and 2 in chlorophyll degradation (Figure 6). A heat map of gene expression reveals that most of the genes involved in chlorophyll synthesis showed upregulation in yellowing leaves, while the genes *HEMF* and *HEMYs*, which are downstream responses of Coproporphyrin III, showed a downregulation trend. During chlorophyll degradation, we found that *PAO* and *RCCR*, which catalyze chlorophyllide a degradation in yellowed leaves, showed an upregulation trend in all developmental periods, leading to the degradation of chlorophyll a synthesis substrates, which might be the reason why chlorophyll a was significantly lower in yellowed leaves compared to normal leaves. In addition, during the chlorophyll cycle, *CAO*, a gene involved in the conversion of chlorophyllide a to chlorophyllide b, showed an overall upregulation in yellowed leaves, which also further reduced the content of chlorophyllide a and increased chlorophyllide b. In summary, the reason for the lower content of chlorophyllide a in yellowed leaves compared with that in normal leaves may be due to chlorophyllide a degradation and the dual effect of conversion to chlorophyllide b, where the enhanced conversion to chlorophyllide b also resulted in a higher content of chlorophyllide b than in normal leaves.

**Figure 7:**
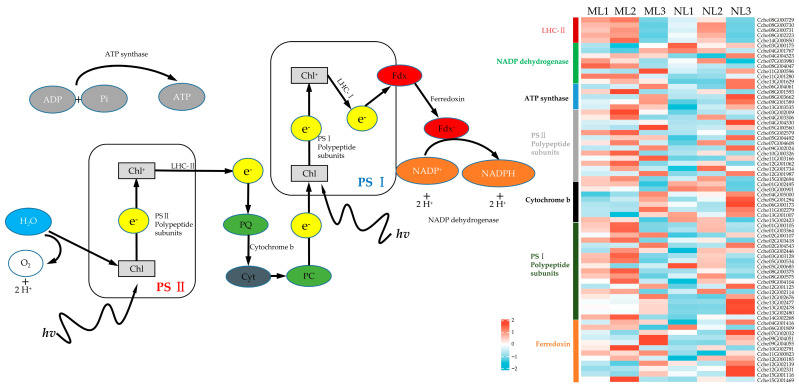
Differential expression of genes related to photosynthesis (light reactions). The expression level was based on FPKM value.

Considering the influence of chlorophyll content changes on light and action, in the expression analysis of photosynthesis-related genes, it was found that in the first two periods, most of the photosynthesis-related genes in the yellowed leaves were expressed at a higher level in the S1 or S2 period. In combination with the trend of changes in the chlorophyll content, the first two periods were mainly the phases of chlorophyll synthesis and the chlorophyll cycle, and the expression of photosynthesis-related genes was relatively high, with the yellowing of the leaves in the maturation stage (Figure 7). The expression of chlorophyll degradation-related genes increased rapidly and showed upregulation relative to normal leaves, while photosynthesis in the mature leaves showed a significant decrease compared to the normal leaves.

### 2.8. Differential Expression Analysis of Genes Related to Carotenoid Synthesis

**Figure 8:**
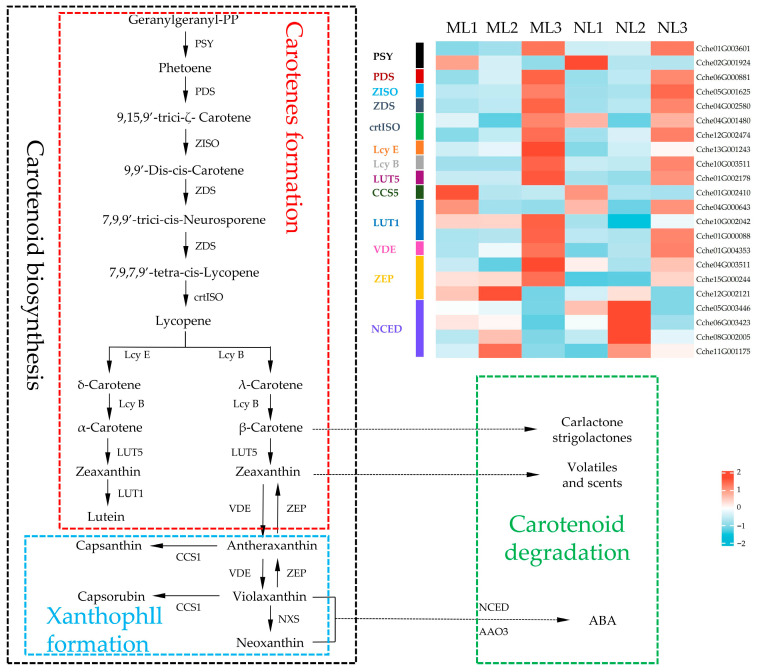
Differential expression of genes related to the carotenoid metabolism pathway. The expression level was based on FPKM value. *PSY*, phytoene synthase; *PDS*, phytoene desaturase; *ZISO*, ζ-carotene isomerase; *ZDS*, ζ-carotene desaturase; *crtISO*, carotenoid isomerase; *Lcy E*, ε-cyclase; *Lcy B*, β-cyclase; *LUT5*, β-hydroxylase; *CCS1*, capsanthin/capsorubin synthase; *LUT1*, ε-cyclase; *VDE*, violaxanthin de-epoxidase; *ZEP*, zeaxanthin epoxidase; *NCED*, 9-cis-epoxycarotenoid dioxygenase.

Carotenoids are involved in plant growth and development and are pigments that influence plant color. It was found that the expression levels of carotenoid synthesis genes in both leaves were lower in the first two periods and increased substantially in the third period, indicating that the accumulation of carotenoids mainly occurred in the later stages of leaf development, while carotenoid degradation-related genes were most highly expressed in the second period, which may be a critical period for the degradation of carotenoids and their conversion into ABA (Figure 8). While comparing the two leaf types in the same period, we found that although most of the genes were slightly upregulated during the carotene formation stage in the yellowed leaves, the downregulation of PSY, as the rate-limiting enzyme for carotenoid synthesis, in the yellowed leaves limited carotenoid synthesis, and the significant upregulation of the *ZEP* genes also blocked xanthophyll formation, resulting in a lower content of lutein-like substances, such as Neoxanthin, in the yellowed leaves than in the normal leaves, which is consistent with the physiological and metabolomic results, and the higher concentration of Neoxanthin may be the reason for the accelerated ABA conversion by the upregulation of the *NCED* gene in normal leaves.

### 2.9. Differential Expression Analysis of Genes Related to Flavonoid Synthesis

**Figure 9:**
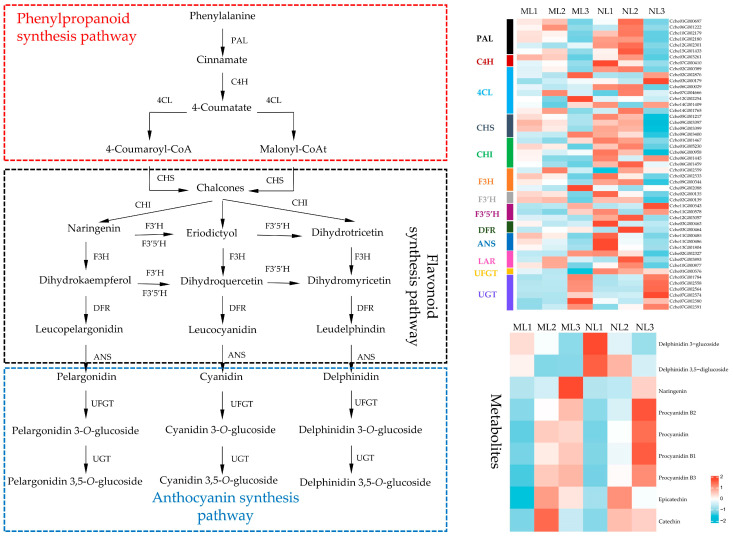
Differential expression of genes related to the anthocyanin metabolism pathway. The expression level was based on FPKM value. *PAL*, phenylalanine ammonia lyase; *C4H*, cinnamate 4-hydroxylase; *4CL*, 4-coumarate: CoA ligase; *CHS*, chalcone synthase; *CHI*, chalcone isomerase; *F3H*, flavanone 3-hydroxylase; *F3′H*, flavonoid-3′-hydroxylase; *F3′5′H*, flavonoid-3′,5′-hydroxylase; *DFR*, dihydroflavonol 4-reductase; *ANS*, anthocyanidin synthase; *UFGT,* UDP-glucose: anthocyanidin 3-O-glucosyltransferase; *UGT,* cyanidin 3-O-rutinoside 5-O-glucosyltransferase.

Anthocyanin biosynthesis is a downstream pathway of the flavonoid synthesis pathway, and systematic analysis of the expression of genes related to the flavonoid synthesis pathway is important for analyzing the effect of anthocyanin biosynthesis on leaf color. A total of 49 differentially expressed candidate genes were identified in this study (Figure 9). Comparing the two leaf types in the same period of time, we found that most of the structural genes in the phenylpropane synthesis pathway and the flavonoid synthesis pathway were downregulated in the yellowed leaves and that the expression of these genes was more active in the first two developmental periods when comparing the two leaf types at different developmental periods. Unlike the upstream pathway, the key genes of the anthocyanin synthesis pathway, *UFGT* and *UGT*, were also downregulated in yellowing leaves, but the active expression of *UGT* was concentrated in the third period. Overall, the downregulation of anthocyanin synthesis-related genes may be the reason why the total anthocyanin content in yellowing leaves was lower than that in normal leaves.

Combined with the metabolomic data, Naringenin, Procyanidin, and delphinidin 3,5-O-diglucoside were detected in the leaves of *Camellia chekiangoleosa*, which showed that geranin, cornflowerin, and delphinidin might be the main types of anthocyanins in the leaves of *Camellia chekiangoleosa*. Delphinidin 3-O-diglucoside and delphinidin 3,5-O-diglucoside were mainly accumulated in the first period, which was consistent with the fact that most of the genes for anthocyanin accumulation were expressed in the first period. Using metabolomic data, we also found that several proanthocyanidins had high relative contents and were abundant in mature leaves, whereas catechins and epicatechins, which constitute proanthocyanidins, accumulated in the first period, providing a source for polymerization into different proanthocyanidins in the later period. Overall, the contents of anthocyanins and proanthocyanidins in yellowed leaves were lower than those in normal leaves, which was consistent with the results of the comparison of measured total anthocyanins.

### 2.10. Analysis of the Co-Expression Regulatory Network for Chlorophyll Content

**Figure 10:**
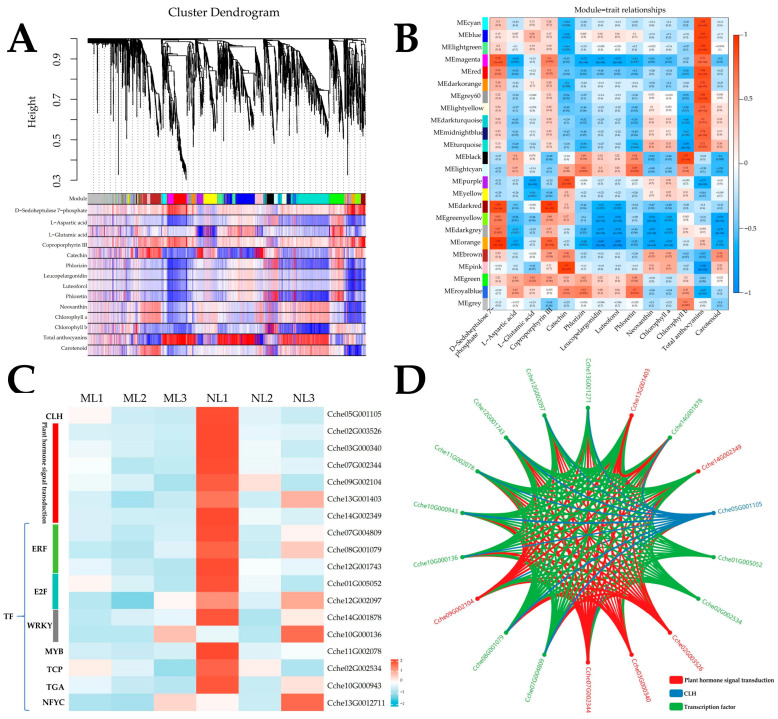
(**A**) Clustering dendrograms of genes. (**B**) Heat map showing the expression profile of each cluster eigengene. (**C**) Heat map of the expression of functional genes and transcription factors in 3 periods of 2 leaf types. (**D**) Co-expression network between the functional genes and transcription factors.

In order to identify the key genes related to chlorophyll differences in normal and mutant leaves and analyze their regulatory networks, all the genes identified in the transcriptome were divided into 24 co-expression modules by co-expression analysis (Figure 10A,B). Among them, the module MEoranger (*p* = 9 × 10^−5^) was significantly associated with chlorophyll a, with a correlation coefficient of −0.74. The module MEblack (*p* = 2 × 10^−6^) and MEturquoise (*p* = 9 × 10^−5^) were significantly associated with chlorophyll b, with correlation coefficients of 0.87 and −0.79, respectively. The module MEorange had a relatively small number of genes (33 genes), followed by module MEblack (61 genes), and the module MEturquoise had the most genes, containing 356 genes. Therefore, we selected the core genes negatively correlated with chlorophyll b synthesis from the module MEturquoise with the largest number of genes. Through KEGG pathway enrichment, we found that this module contains a structural gene (chlorophyllase) *CLH* (Cche05G001105) that plays an important role in chlorophyll metabolism and is responsible for the conversion of chlorophyll a and b to chlorophyllide a and b. This gene has a higher expression in the S1 stage of the leaf, and its expression is higher in the normal part of the leaf. The higher expression of this gene in the S1 stage of the leaf and the normal part of the leaf suggests that the elevated chlorophyll b content in the yellowed leaves may be caused by the negative regulation of this gene for the chlorophyll content. The most enriched gene in the module MEturquoise was plant hormone signal transduction (6), suggesting that plant hormones play an important role in the variation in the chlorophyll b content. In addition, three ERFs, two E2Fs, two WRKYs, and transcription factor genes, such as MYB, TPC, TGA, NFYC, etc., were also found in this module (Figure 10C). Through the weights and correlations of the above genes, the co-expression regulatory network was constructed with CLH as the hub gene, reflecting the regulatory role of related transcription factors and plant hormone signal transduction genes on the expression of *CLH* genes (Figure 10D). Meanwhile, in the module MEblack, we enriched an *ELIP* (early light-induced protein 1) gene, which was more than a thousand times more expressed in the third stage of leaf development than in the first two stages and highly expressed in the yellowed portion of leaves, which may play an important role in the formation of yellowed leaves.

### 2.11. Real-Time Quantitative Validation

**Figure 11:**
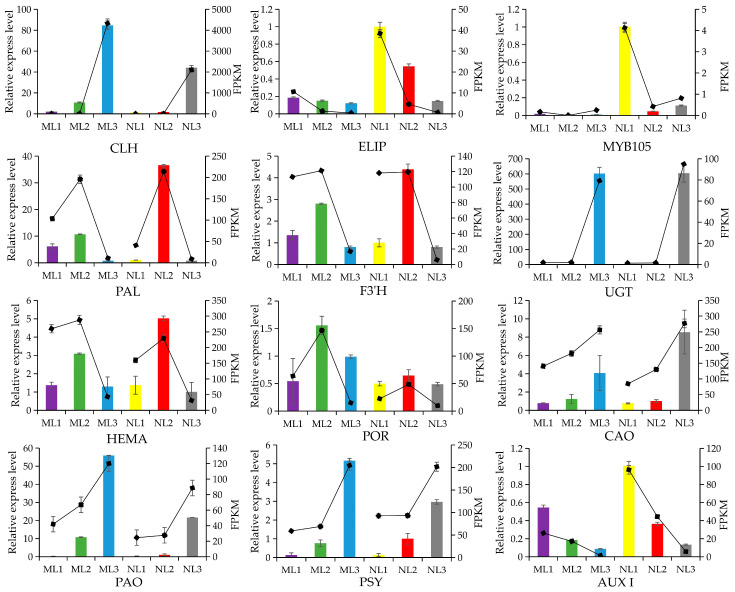
Relative expression levels and FPKM of 12 genes. qRT–PCR results are shown in the column configuration, and FPKM results are displayed as line charts.

The synthesis of chlorophyll, carotenoids, and anthocyanins in leaves may all have an impact on the color change of leaves, from which 12 structural genes related to pigment synthesis were selected for qRT-PCR validation, and it was found that the experimental results were basically consistent with the trend of FPKM value changes in the transcriptome (Figure 11). Among them, the genes related to chlorophyll a degradation all reached the highest in the third period, and the expression of the PAO gene in the mutant leaves was much higher than that in the normal leaves, which preliminarily confirmed that the degradation of chlorophyll a led to the decrease in chlorophyll a content and demonstrated the scientific validity of the sampling and sequencing in this study.

## 3. Discussion

In this study, the mutant *Camellia chekiangoleosa* clone LK34 was identified and characterized as having both normal leaves and yellowed leaves that underwent bud mutation. Upon observation, this yellowing was found to start from the petiole and spread along the leaf veins as the leaf developed, which is different from most irregular mutations, such as whole-leaf yellowing, spotting, and streaking in nature, for example, the presence of whole-leaf yellowing [25,26] and streaking yellowing in Ginkgo [1] and the complete yellowing of the leaves in poplar and zoysia, among others [19,27]. Not only that, the yellowing of the yellowed leaves of the mutant LK34 had already occurred during the period of young leaves and spread stably with leaf development, which was different from the leaves of Anji white tea that showed yellow or white at low temperatures and recovered their green color with warming, suggesting that the leaf phenotype was not caused by environmental factors [20], and the green part of the yellowed leaves was consistent with the change in the normal leaves, which indicated that the yellowing was not caused by leaf senescence, which was also corroborated by the analysis of senescence-related gene expression. Therefore, by comparing normal and mutant leaves at different developmental periods in the *Camellia chekiangoleosa* mutant plant LK34, we were able to systematically study the causes of leaf yellowing and the effects on growth.

In view of this, through the observation of cell structure and determination of pigment content, the present study found that the alteration of the chloroplast structure and a drastic reduction in chlorophyll a/chlorophyll b were the direct causes of the formation of the yellowing mutation. Under transmission electron microscopy observation, the chloroplasts of normal leaf-colored plants were ellipsoidal or subellipsoidal, containing abundant, well-defined endomembrane systems and densely textured tissues, with distinct basidiomata and basidiomatous lamellae in the endomembrane systems [28]. In contrast, the cells in the yellowed portion of the mutant leaf were clearly and severely altered in their chloroplast structure compared to the normal leaves, with no cyst-like stacking structure observed in the chloroplasts of the majority of the cells, no obvious cyst-like membrane segregation, and a lack of starch granules commonly found in normal chloroplasts, with a larger area of the organelle instead occupied by vesicles and osmiophilic granules. Similar phenomena have been found in earlier excavated leaf color mutants of rice, cabbage, and oilseed rape [22,29,30]. Among the closely related species, Ma et al. found that the number of chloroplasts in the yellow mutant leaves of Anji white tea ‘Yinghong No. 9’ was significantly reduced compared with that in normal green leaves, and there was a lack of intact cyst-like membranes within the chloroplasts, a reduction in the number of cysts, and no basal stacks [31]. Similar to these studies in which the degradation of the chloroplast structure was often accompanied by a decrease in chlorophyll content, the content of chlorophyll in the yellowed portion of mature mutant leaves in the present study was about one-third of that in normal leaves, whereas the content of carotenoids was slightly higher than that in normal leaves, and the difference in anthocyanins was not significant, suggesting that the substantial decrease in chlorophyll content was the main reason for the loss of greenness in some portions of the leaves. However, the contents of chlorophyll a and chlorophyll b are decreased in most yellowing mutants. For example, the yellowing leaves in Ginkgo had lower chlorophyll a and chlorophyll b than normal leaves, and the elevation of carotenoids maintained the yellow color after the loss of greening [1]. Whereas, while a decrease in chlorophyll a occurred in the yellowed portion of LK34 in the present study, chlorophyll b was increased dramatically, which explained the leaves did not completely lose their greenness and turned white in the case of the decline of the dominant yellow carotenoids, and it was the rise in the yellow-green chlorophyll b content that maintained the yellow color and formed the yellowed portion of the mutant leaves.

We further analyzed the intrinsic mechanism of leaf yellowing by comparing transcriptome and co-expression and found that the high expression of *PAO*, *RCCR*, and *CAO* genes in the yellowed leaves was the dominant reason for the lower content of chlorophyll a compared with that in normal leaves. The former two catalyzed the degradation of chlorophyll a, while the latter catalyzed the conversion of chlorophyll a to chlorophyll b. At the same time, the low expression of the *CLH* gene was the dominant reason for the higher content of chlorophyll b. The co-expression module of this gene showed a significant negative correlation with the content of chlorophyll b. In the study of the poplar golden leaf mutant, the researchers concluded that the high expression of the chlorophyll-degrading gene *PAO* led to a significant reduction in chlorophyll by degradation [27]. Unlike that study, the present study found that in addition to the high expression of genes involved in chlorophyll degradation, the genes involved in the conversion of chlorophyll a to chlorophyll b were equally high, which is consistent with the decrease in the content of chlorophyll a but the increase in the content of chlorophyll b in the mutant leaves. Moreover, it has been shown that *PAO* catalyzes the oxidation of demagnetized chlorophyll a at the same time [32], and Dong et al. found, through silencing the *RCCR* gene in tobacco, that this gene has a promoting regulatory effect on the expression of *CLH* and *PAO* genes, further promoting the degradation of chlorophyll a. Therefore, in this study, RCCR in mutant leaves is likely to promote the expression of *PAO* genes and increase the degradation of chlorophyll a [33]. On the other hand, it has long been shown that the CAO-catalyzed conversion of chlorophyll a to chlorophyll b is a key regulatory step in the chlorophyll metabolic pathway [34]. However, *CLH* is considered to be the first enzyme in the chlorophyll metabolic pathway. It was found that the virus-induced silencing of *CHLD* and *CHLI* (CLH and magnesium chelatase subunits D and I) in pea (*Pisum sativum*) resulted in a yellow-leaf phenotype, undeveloped membranes in the transgenic plants, structural changes in the chloroplasts, and aberrant antenna complexes [35]. The results were the same as those observed cytologically in this study [36]. Therefore, the present study summarizes the conjecture of the regulatory mechanism of yellowing leaves by the *RCCR* gene promoting *PAO* expression to promote chlorophyll a degradation, combined with the conversion of chlorophyll a to chlorophyll b catalyzed by *CAO*, which leads to the reduction of the chlorophyll a content, while the *CLH* gene expression under the regulation of transcription factors is lower than that of the normal leaves, maintaining a higher content of chlorophyll b and making the leaves partially appear yellow. It is necessary to further verify and analyze the temporal and spatial expression sequence of leaf yellowing.

In addition to the above findings, this study also found that the chloroplast structure, pigment content, and photosynthesis have an interactive effect. In this study, among the genes that were differentially expressed in both normal and mutant leaves in three periods, we found that the *PsaB* gene, which is involved in photosynthesis PSII, the *PsbB*, which is involved in electron transfer in photosynthesis PSI, and the subunit PetB of Cytochrome b complex were all downregulated in the yellowed part, which was the same as the lower photosynthesis in the mutant leaves than in the normal leaves. Among them, PsaB and PsbB are the key enzymes for transferring electrons in PSII and PSI, respectively. It was also found that when overexpressing *OsGLK* in rice, the expression of *psbA*, *psaA*, and *rbcL* genes encoding chloroplast proteins was significantly upregulated, which prevented the formation of chloroplasts and rendered the leaves stagnant green [37]. The Cytochrome b6f complex, on the other hand, is the electron-transfer chain linking PSII and PSI. It catalyzes the rate-limiting step in linear electron transfer (LET) and plays a key role as a redox-sensing center in regulating light harvesting, electron transfer, and photosynthetic gene expression [38]. Reflecting the fact that the blockage of photosynthesis also has an effect on the formation of the chloroplast structure, the downregulation of photosynthesis-related genes in the leaves in the present study may account for the differences in the chloroplast structure of the yellowed portion of the chloroplasts from the normal leaves. Chlorophyll, as a core pigment involved in the light reaction of photosynthesis, and carotenoids not only absorb and transmit light energy but also avoid chlorophyll degradation, and the reduction of carotenoid content in yellowed leaves also has an important effect on the acceleration of chlorophyll degradation. In the carotenoid synthesis pathway, this study found that the *PSY* gene may limit subsequent carotenoid synthesis [39], whereas the upregulation of the *ZEP* gene blocked carotenoid synthesis, which was verified in Arabidopsis, where the carotenoid content in the mutant lacking *ZEP* was 6-fold higher than that in the wild type [40]. Thus, the genetically controlled decrease in the chlorophyll and carotenoid content is the main reason for the decrease in photosynthesis in mutant leaves. In addition, for the study of flavonoid metabolites and synthesis pathways, the main anthocyanin types and flavonoid metabolites in the leaves were found, but the difference in anthocyanins in the two types of leaves was less, which indicates that flavonoids did not have a major effect on the mutation and photosynthesis in *Camellia chekiangoleosa* leaves, but the study shows that flavonoids can be involved in apoptosis pathways through different mechanisms, for example, the regulation of apoptosis-related proteins, as transcriptional transducers or activators, through mitochondria-mediated or ROS-mediated apoptotic pathways [41]. Flavonoids, including anthocyanins, also have a variety of physiological functions, such as antioxidant and drought resistance [42,43].

Light and pigment contents are critical for photosynthetic efficiency, and normal leaves with higher photosynthetic pigments, chlorophyll, and carotenoids in this study also had higher photosynthesis, with a higher maximum photosynthetic rate, photosynthetic efficiency, light compensation point, and stomatal conductance than the mutant leaves, similar to the findings of Si et al. and Viljevac et al. in rice and cherry, which both reflect the fact that the mutant leaves have worse photosynthetic characteristics [21,44]. It was shown that leaves of varieties with excellent photosynthetic characteristics in the oil-tea *camellia* species have higher adaptive capacity, higher photosynthetic rates, and lower consumption of photosynthetic products. Their higher acclimatization ability was manifested in a higher apparent quantum efficiency and lower light compensation point under low photosynthetically active radiation conditions (a phenomenon found in this study), and higher carboxylation efficiency and lower CO_2_ compensation point in a low CO_2_ concentration environment. In contrast, the reduction in the Cytochrome b6f complex in photosynthesis system II in the mutant leaves, which was manifested in the reduction in the chlorophyll a/b ratio as well as the reduction in the total chlorophyll content, may be an adaptation mechanism of the mutant leaves to the environment [21].

## 4. Materials and Methods

### 4.1. Materials

The test materials were from the national oil-tea camellia plant Germplasm Gene Bank (Nanchang, China) (28.91° N, 115.65° E, and average altitude 30 m), and an eight-year-old *C. chekiangoleosa* variety ‘Diecui Liujin’ (accession number: 20230583) was selected. Three periods were selected from young leaf formation to leaf maturity—10 DALFs (days after initial leaf formation, S1), 50 DALFs (S2), and 90 DALFs (S3)—for collection, and the leaves entered the maturity stage at S3. Three different orientations of leaves in the same period were selected as biological replicates, with 10 leaves in each replicate, and the normal leaves (NLs, normal leaves from the original plant) and the yellowed portion of the mutant leaves (MLs, mutant leaves with normal parts removed) were stored at −8 °C.

### 4.2. Transmission Electron Microscopic Observation of Leaf Cell Structure

① Sampling and fixation: Mature NLs and MLs were used as the sampling sites, respectively, to minimize mechanical injuries, such as pulling, contusion, and extrusion. The sampling was completed within 1–3 min, and the sampled tissues were 1 mm in size. A Petri dish with an electron microscope fixative (Servicebio, Wuhan, China, G1102) was prepared in advance before sampling, and the small tissue blocks were put into the Petri dish immediately after removal from the body and then cut into 1 mm small tissue blocks with a scalpel in the fixative of the Petri dish. Then, the small tissue block was transferred to an EP tube containing a new electron microscope fixative to continue fixation, vacuum pumped until it reached the bottom, and left at room temperature for 2 h, after which it was placed in fixed storage at 4 °C and transported. The sample was rinsed three times, each time for 15 min, with 0.1 M phosphate buffer PB (PH 7.4). ② Post-fixation: 0.1 M phosphate buffer PB (PH 7.4) was prepared with 1% osmiobutyric acid fixed at room temperature and protected from light for 7 h. A total of 0.1 M phosphate buffer PB (PH 7.4) was fixed at room temperature. A total of 0.1 M phosphate buffer PB (PH 7.4) was rinsed 3 times, each time for 15 min. ③ Room-temperature dehydration: Tissues were sequentially subjected to 30%–50%–70%–80%–95%–100% alcohol upstream dehydration, each time for 1 h. Anhydrous ethanol/acetone = 3:1 for 0.5 h. ④ Anhydrous ethanol/acetone = 1:1 for 0.5 h, anhydrous ethanol/acetone = 1:3 for 0.5 h, and acetone for 1 h. Each sample was embedded at 37 °C for 5–8 h. Pure 812 embedding agent was poured into the embedding plate, and the sample was inserted into the embedding plate and baked in an oven at 37 °C overnight. ⑤ Polymerization: The embedding plate was placed in an oven at 60 °C to polymerize for 48 h, and the resin block was taken out as a spare. ⑥ Ultrathin sectioning: The resin block was subjected to ultrathin sectioning in an ultrathin sectioning machine (Leica, Wetzlar, Germany, Leica UC7) at 60–80 nm, and the samples were sliced through a 150-mesh copper mesh with a square Chinese membrane. ⑦ Staining: Copper mesh was stained with 2% uranyl acetate saturated alcohol solution for 8 min, washed with 70% alcohol 3 times, washed with ultrapure water 3 times, stained with 2.6% lead citrate in carbon dioxide solution for 8 min, washed with ultrapure water 3 times, and drained on filter paper. Copper mesh slices were put into the copper mesh box and dried at room temperature overnight. ⑧ A transmission electron microscope (HITACHI, Tokyo, Japan, HT7800/HT7700) was used for observation, and images were collected for analysis [45].

### 4.3. Determination of Photosynthetic Indicators for Two Types of Leaves

In normal-growing 8-year-old *Camellia chekiangoleosa* ‘DieCui LiuJin’, a total of six mature leaves from the upper, middle, and lower positions of normal and mutant plants were selected for determination as follows: Light response curve determination: using an LI-6400 XT portable photosynthesizer (LI-COR, Lincoln, NE, USA), the LED red and blue leaf chambers were selected, the CO_2_ concentration was set to 400 μmol·s^−1^, the flow rate was set to 500 μmol·s^−1^, the light intensity gradient was set to 2100, 1800, 1500, 1200, 900, 600, 300, 200, 100, 50, 25, 10, and 0 μmol·m^−2^·s^−1^, and the light response curves of the mutant and normal leaves were determined between 9:00 and 11:00. Measurement of gas exchange parameters: The LED red and blue leaf chambers were selected, the CO_2_ concentration was set to 400 μmol·s^−1^, the flow rate was set to 500 μmol·s^−1^, and the light intensity was set to 1000 μmol·m^−2^·s^−1^, and the gas exchange parameters of the mutant and normal leaves were measured between 9:00 and 11:00 [25].

### 4.4. Detection of Non-Targeted Metabolomes

NLs and MLs were taken as test materials, and the experimental steps were as follows: ① 80 mg of a sample was weighed, and 20 μL of internal standard (L-2-chlorophenylalanine, 0.06 mg/mL; methanol configuration) and 1 mL of methanol–water (*v:v* = 7:3) were added; ② Two small steel beads were added before the sample was pre-cooled at −20 °C for 2 min and added to the mill (60 Hz, 2 min); ③ The sample was ultrasonicated in an ice-water bath. Extraction was performed for 30 min in an ice-water bath, and the sample was allowed to stand overnight at −20 °C. ④ Centrifugation was performed for 10 min (13,000 rpm, 4 °C), and 150 μL of the supernatant was aspirated with a syringe, filtered through a 0.22 μm organic-phase needle-well filter, and then transferred to an LC injection vial and stored at −80 °C until LC-MS analysis. ⑤ Quality control (QC) samples were prepared by mixing the extracts of all samples in equal volume. The analytical instrument for the experiments was a liquid–mass spectrometry (LMS) system consisting of an ACQUITY UPLC I-Class plus ultra-high-performance liquid chromatography tandem with a QE high-resolution mass spectrometer (Waters, Milford, MA, USA). The chromatographic conditions were as follows: column: ACQUITY UPLC HSS T3 (100 mm × 2.1 mm, 1.8 um); column temperature: 45 °C; mobile phases: A-water (containing 0.1% formic acid) and B-acetonitrile (containing 0.1% formic acid); flow rate: 0.35 mL/min; injection volume: 2 μL; and mass spectrometry (MS) conditions: ESI (electrospray ionization) ion source. The mass spectrometry signals were acquired in positive- and negative-ion scanning modes [27].

### 4.5. Measurement of Leaf Physiological Indexes

NLs and MLs of the S1, S2, and S3 stages were taken as experimental materials, respectively, and ethanol acetone extraction was used to determine the content of chlorophyll a and chlorophyll b using the colorimetric method [46]; the carotenoid content using the colorimetric method [47]; and the content of phycocyanin using UV spectrophotometry [48].

### 4.6. Transcriptome Sequencing and Differential Analysis

Total RNA was extracted from 18 samples from three stages of three replicates of two leaves using the RNAprepPurePlantKit80 (TIAGEN, Beijing, China) kit [49]. The extent of RNA degradation and contamination was detected by agarose gel electrophoresis. An Agilent 2100 Bioanalyzer (Agilent Technologies, Santa Clara, CA, USA) was used for RNA quality modeling, and samples with an RNA integrity index (RIN) ≥ 7 were sequenced. The Illumina Sequencing Platform was used to obtain second-generation sequencing (NGS) data and calculate the raw error rate (%), Phred score (Q30), GC content (%), and clean data rate (%) to assess the quality of the raw data. Raw data (raw reads) were processed using Trimmomatic [50]. The reads containing ploy-N and the low quality reads were removed to obtain clean reads. Then, the clean reads were mapped to the reference genome using hisat2 [51]. The FPKM value of each gene was calculated using cufflinks [52], and the read counts of each gene were obtained by htseq-count [53]. DEGs were identified using the DESeq R package version 1.38.0 functions estimateSizeFactors and nbinomTest. A *p*-value < 0.05 and foldChange > 2 or foldChange < 0.5 were set as the threshold for significantly differential expression [54]. Hierarchical cluster analysis of DEGs was performed to explore gene expression patterns. GO enrichment and KEGG [55] pathway enrichment analyses of DEGs were, respectively, performed using R based on the hypergeometric distribution.

The FPKM and read count values of each transcript (protein_coding) were calculated using bowtie2 (http://bowtie-bio.sourceforge.net/bowtie2/index.shtml) and eXpress (http://bio.math.berkeley.edu/eXpress/) [56,57]. DEGs were identified using the DESeq functions estimateSizeFactors and nbinomTest. A *p*-value < 0.05 and foldChange > 2 or foldChange < 0.5 were set as the thresholds for significantly differential expression. Hierarchical cluster analysis of DEGs was performed to explore the transcript expression patterns. GO enrichment and pathway enrichment analyses of DEGs were, respectively, performed using R based on the hypergeometric distribution.

### 4.7. Weighted Co-Expression Analysis and Key Gene Identification

The R software package WGCNA version 1.61 was used to analyze the co-expression of genes with FPKM: ≥ 1 or a variation of FPKM: cv ≥ 0.5. The default network structure and module detection methods included an unsigned topological overlap matrix (TOM) with a power β of 24, the maximum number of modules was 50, the minimum module size was 30, and the branches were merged at a height of 0.25. The modulus eigenvalue (ME, the first principal component of a given modulus) of each module was calculated and used to test the correlation with the oil content. The module membership (MM) metric was determined by correlating the expression profile of a gene with the ME of its resident module, and this was highly correlated with the intramodule connectivity (K.in). Then, the KEGG enrichment method was used to mine pigment synthesis and regulation-related genes (the transcription factors). Finally, based on MM and K.in, Cytoscape version 3.9.1 [58] software was used to draw the regulatory network of transcription factors and structural genes.

### 4.8. Real-Time Quantitative Measurement

The FPKM values of genes obtained from the sequencing of 18 samples were compared, and stable housekeeping genes were selected for initial validation using qRT-PCR. Nine structural genes related to pigment synthesis and photosynthesis in leaves were randomly selected for qRT-PCR experiments. Three technical replicates were performed for each sample. The 20 μL qRT-PCR system consisted of 7.8 μL ddH_2_O, 0.6 μL primers, 1 μL cDNA, and 10 μL EvaGreen2x qPCR parent (Canada Applied Biomaterials, Richmond, BC, Canada). The viiATM7 system (Applied Biosystems, Foster City, CA, USA) was used according to the following procedure: initial denaturation at 95 °C for 10 min and denaturation at 95 °C for 15 s, followed by 45 annealing cycles and the assessment of primer specificity by dissociation curve analysis at 60 °C. The comparative cycling threshold (Ct) method and the 2^−ΔΔCT^ formula [59] were used.

## 5. Conclusions

In this study, changes in the yellowing leaves of the *Camellia chekiangoleosa* mutant cultivar ‘Diecui Liujin’ were systematically analyzed at the cellular, physiological, and transcriptional levels. The results show that in the mutant leaves, the high expression of genes catalyzing the conversion of chlorophyll a to chlorophyll b (*PAO*, *RCCR*) and chlorophyll a degradation (*CAO*) led to a decrease in the chlorophyll a content, and the former, together with the low expression of the *CLH* gene, led to an increase in the yellowish-green chlorophyll b content, which was the intrinsic cause of yellowing in the mutant leaves. These results provide insights into the mechanism of leaf color variation, and from these insights, the theoretical underpinning for the utilization and selective breeding of camellia chekiangoleosa with characterized leaf color may be developed.

## Data Availability

Data will be made available on request.

## Supplementary Materials

The supporting information can be downloaded at: https://www.mdpi.com/article/10.3390/ijms26010132/s1.
