# Supplementary material for: Cytological, Physiological, and Transcriptome Analysis of Leaf-Yellowing Mutant in Camellia chekiangoleosa
Source: Int J Mol Sci. 2024 Dec 27;26(1):132. doi: 10.3390/ijms26010132 (PMC11719897; doi:10.3390/ijms26010132)
Supplement: Supplementary file 1 [file ijms-26-00132-s001.zip › ijms-3380279-supplementary.pdf]

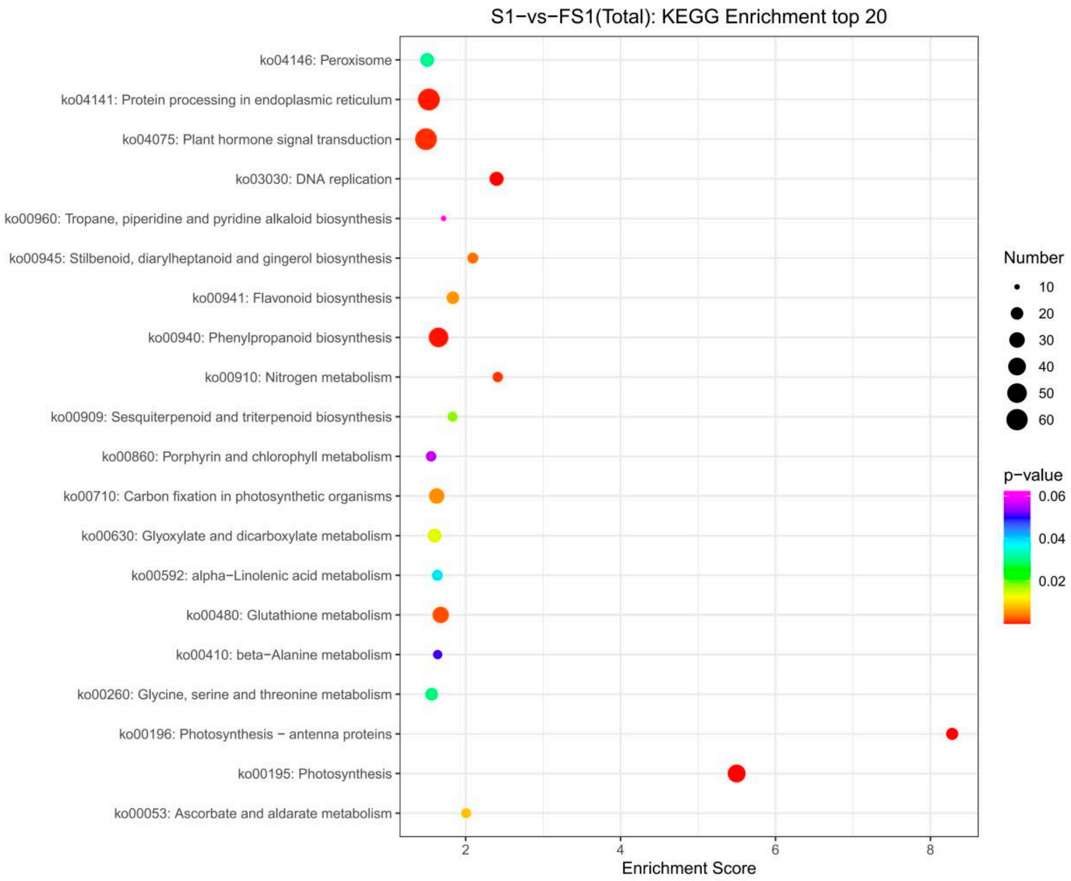

Figure S1. Kegg annotation of the differential genes between the two leaves in the S1 period (TOP 20)

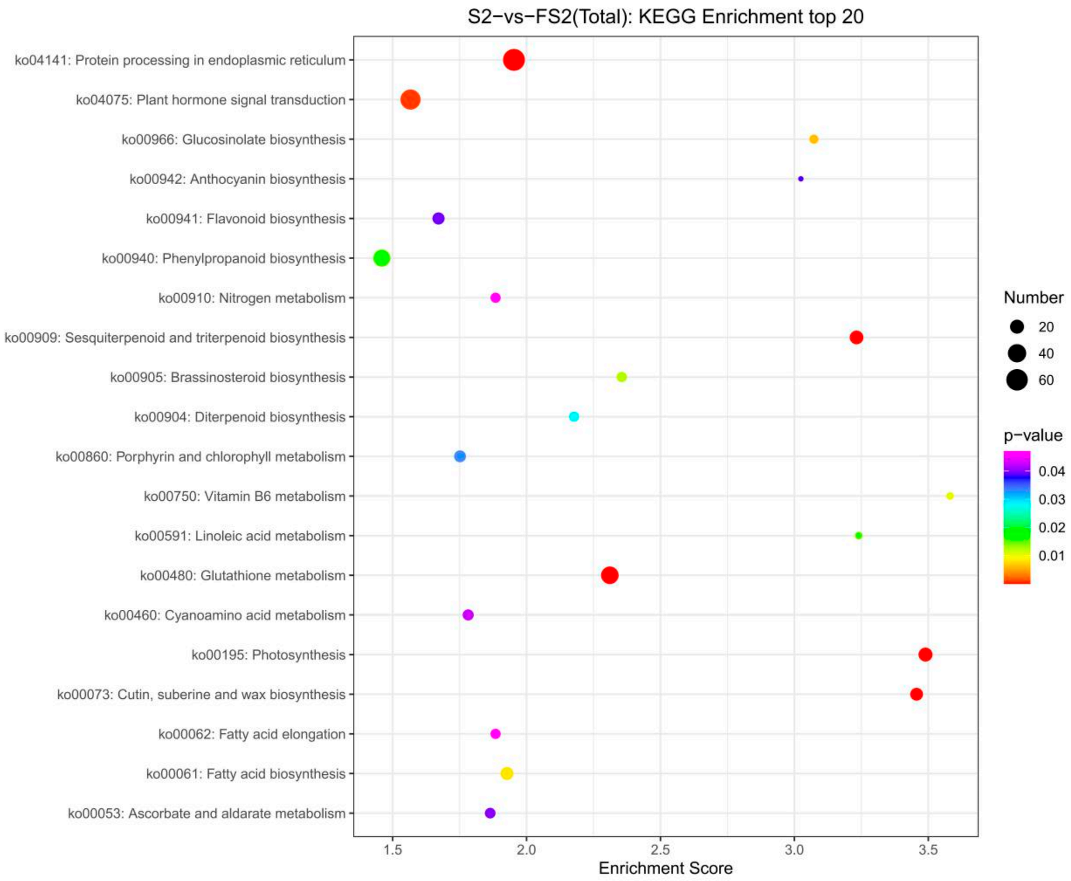

Figure S2. Kegg annotation of the differential genes between the two leaves in the S2 period (TOP 20)

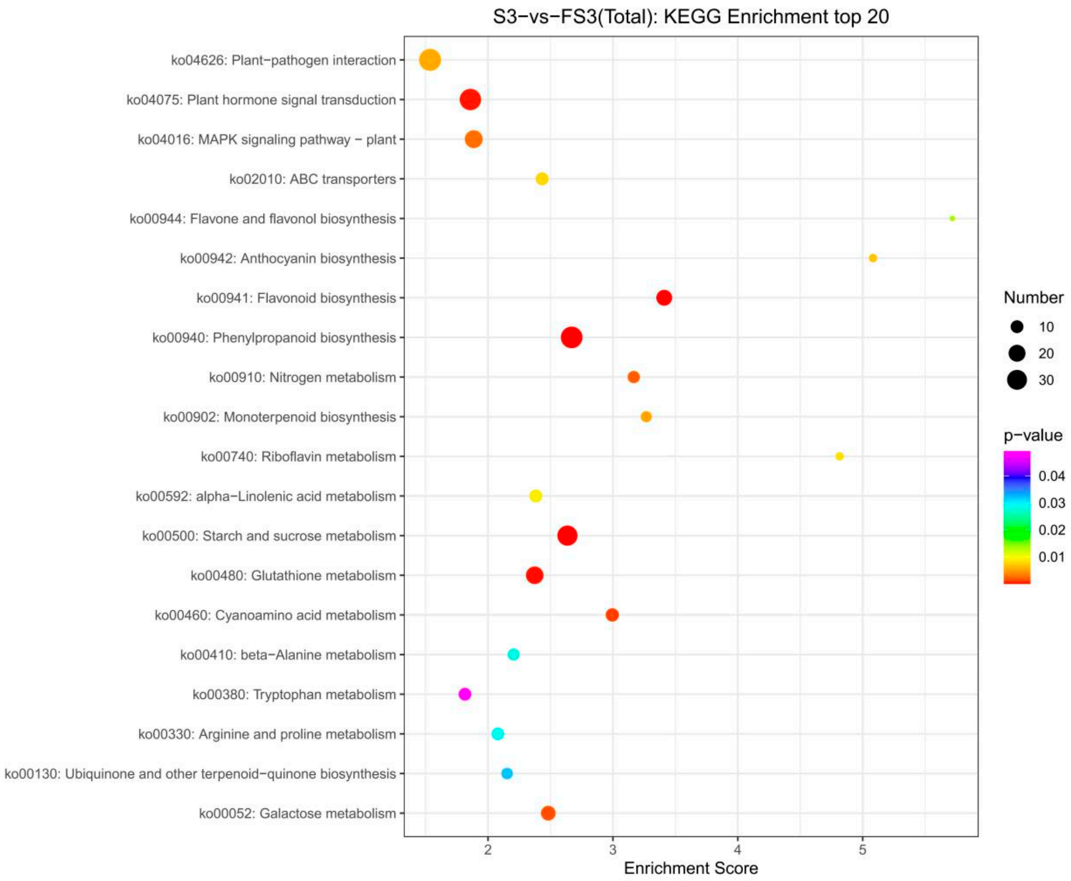

Figure S3. Kegg annotation of the differential genes between the two leaves in the S3 period (TOP 20)

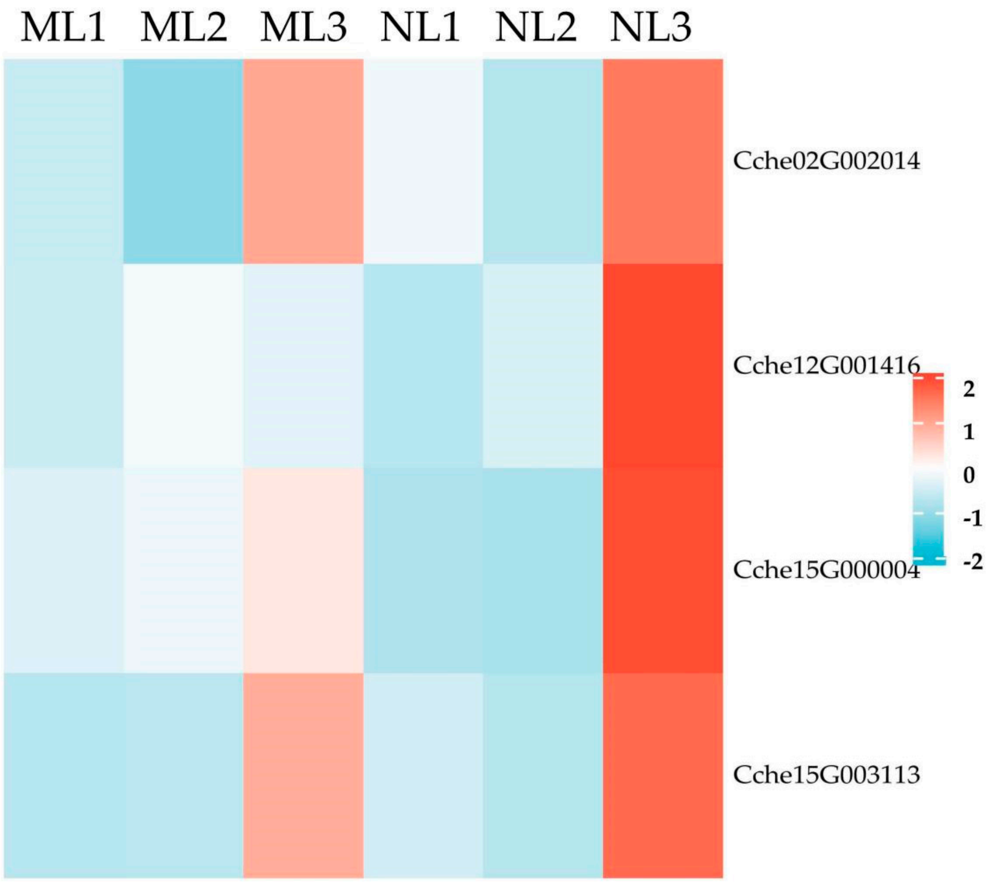

Figure S4. Expression heatmap of 4 SAGs
